# Poly(3‐hexylthiophene)s Functionalized with N‐Heterocyclic Carbenes as Robust and Conductive Ligands for the Stabilization of Gold Nanoparticles

**DOI:** 10.1002/anie.202012216

**Published:** 2020-12-29

**Authors:** Ningwei Sun, Shi‐Tong Zhang, Frank Simon, Anja Maria Steiner, Jonas Schubert, Yixuan Du, Zhi Qiao, Andreas Fery, Franziska Lissel

**Affiliations:** ^1^ Institute of Macromolecular Chemistry Leibniz Institute of Polymer Research Hohe Strasse 6 01069 Dresden Germany; ^2^ Institute for Physical Chemistry and Polymer Physics Leibniz Institute of Polymer Research Hohe Strasse 6 01069 Dresden Germany; ^3^ State Key Laboratory of Supramolecular Structures and Materials College of Chemistry Jilin University Changchun 130012 China; ^4^ Technische Universität Dresden Mommsenstrasse 4 01064 Dresden Germany

**Keywords:** chain growth polymerization, conjugated polymers, gold nanoparticles, hybrid materials, N-heteroyclic carbenes

## Abstract

Recently, N‐heterocyclic carbenes (NHCs) are explored as anchor groups to bind organic ligands to colloidal gold (i.e. gold nanoparticles, Au NPs), yet these efforts are confined to non‐conjugated ligands so far—that is, focused solely on exploiting the stability aspect. Using NHCs to link Au NPs and electronically active organic components, for example, conjugated polymers (CPs), will allow capitalizing on both the stability as well as the inherent conductivity of the NHC anchors. Here, we report three types of Br‐NHC‐Au‐X (X=Cl, Br) complexes, which, when used as starting points for Kumada polymerizations, yield regioregular poly(3‐hexylthiophenes)‐NHC‐Au (P3HTs‐NHC‐Au) with narrow molecular weight distributions. The corresponding NPs are obtained via direct reduction and show excellent thermal as well as redox stability. The NHC anchors enable electron delocalization over the gold/CP interface, resulting in an improved electrochromic response behavior in comparison with P3HT‐NHC‐Au.

## Graphical abstract

Br‐NHC‐Au^I^ complexes were designed as starting points of Kumada polymerizations to grow regioregular poly(3‐hexylthiophene)s‐NHC‐Au^I^, which were reduced to afford P3HT‐NHC@Au NPs. In addition to excellent thermal as well as redox stability, the corresponding NPs show improved charge transfer behavior due to the effective electron delocalization over the gold/P3HT interface benefiting from the robust and conductive NHC linkages.

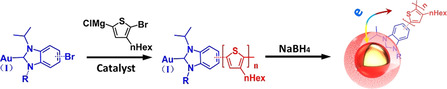

Gold nanoparticles (Au NPs) have unique surface plasmonic resonances (SPR) enabling a broad variety of promising optical applications in sensors and imaging.^[1]^ To stabilize the Au NPs and prevent irreversible aggregation, protecting ligands are required, which usually carry one or more functional groups (anchor groups) with a high affinity for gold. So far, thiols are the most ubiquitously used anchor group, yet the thermal instability and comparably low conductance of the S−Au contact limit the applications of the resulting Au NPs.^[2]^ Recently, NHCs have been receiving much interest in surface chemistry as a versatile alternative to sulfur‐based ligands.^[3]^ NHCs give access to a rich synthetic chemistry and are easily structurally modified. The NHC−Au linkage shows excellent stability under conditions that destroy the S−Au bond (e.g. high temperature, variable pH, and electrochemical redox).^[4]^ In addition, the NHC−Au bond is highly conductive and was used in molecular electronics to increase the performance of transistors.^[5]^ On the other hand, charge‐conducting conjugated polymers (CPs) are key components for various electronic devices, yet so far, most reported polymeric NP ligands consist of non‐conjugated polymers bound to coinage metals via non‐conductive anchor groups. In the few cases when a CP was bound to NPs, it was paired with an insulating anchor group,^[6]^ while conductive anchors such as NHCs have so far only been used in conjunction with nonconductive polymers. Using NHCs as robust and conductive anchors to bind CPs to Au NPs would yield a new class of electronically functional hybrid materials. The combination of CPs and Au NPs could pave the way to electronically functional hybrid materials with multiple functions beyond the sum of the single components, for example, by combining the unique properties of Au NPs and polymer semiconductors.^[7]^ The highly conductive NHC anchor will increase the electron transfer at the Au NP/CP interface, and furthermore modify the work function of the metal.^[8]^

To date, there are two common approaches to synthesize NHC‐functionalized Au NPs, namely top‐down and bottom‐up methods.[^3a^, ^3b^, ^3c^, ^3d^, ^9^] The former one directly adds free NHCs to a pre‐existing Au NP surface, and requires conditions without labile protons. Due to this significant limitation, it is extraordinarily difficult to synthesize CPs functionalized with free NHCs. The latter one reduces an NHC−Au complex to form NHC‐based Au NPs. The NHC−Au bond has a high bond energy and is therefore stable, and recently bipodal NHCs were designed to further improve the stability of the resulting Au NPs (Figure 1).^[4b]^ Using direct reduction, water‐soluble PEG‐NHC@Au NP containing polyethyleneglycol (PEG) ligands was prepared for potential applications in biological media.^[10]^

**Figure 1:**
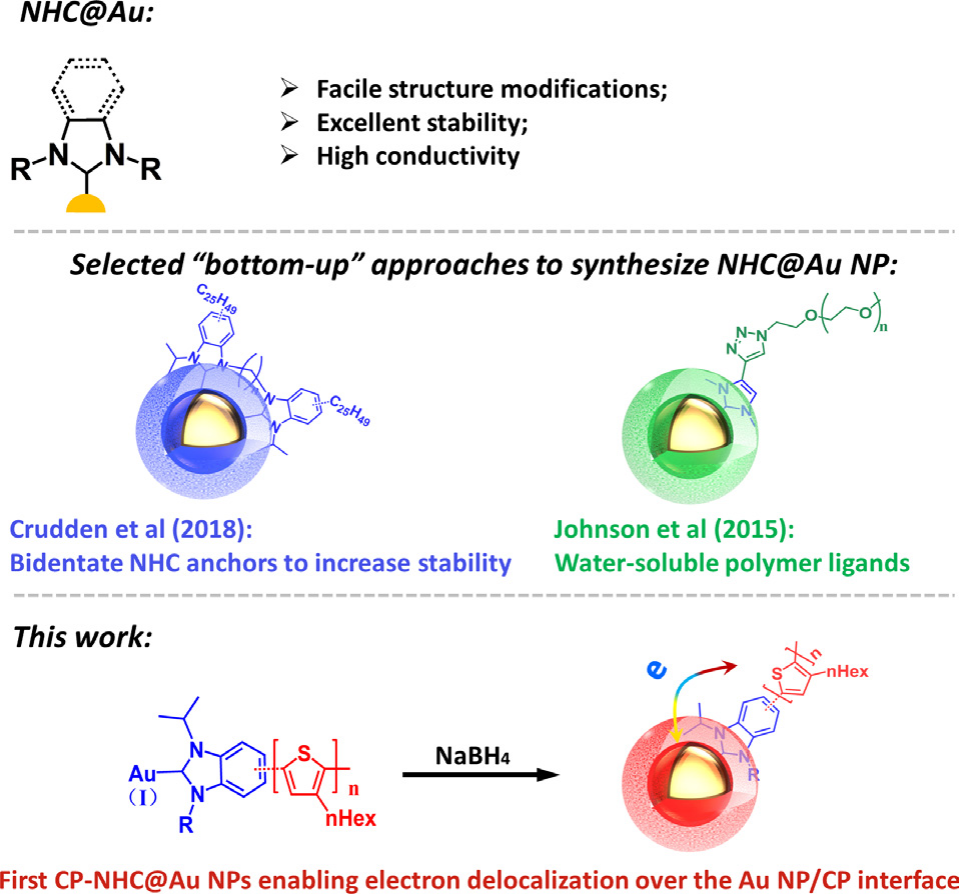
Top: advantages of the NHC@Au anchor; middle: examples of prior NHC@Au NPs reports using electronically passive polymers as organic ligands;[^4b^, ^10^] bottom: conjugated P3HT functionalized with novel NHC−Au complexes yields P3HT‐NHC@Au NP composites.

Therefore, the bottom‐up approach seems a feasible strategy to access CP‐NHC@AuNP composites. The precursor of such composites would be CPs functionalized with an NHC−Au group, and to obtain them, externally initiated Kumada polymerization is a powerful approach: Kumada polymerization allows to selectively grow polythiophenes, one of the most important CPs, from chosen initiators, yielding mono‐functionalized polythiophenes.^[11]^ Moreover, the resulting polythiophenes, for example, poly(3‐hexyl‐thiophene) (P3HT), are regioregular, which is beneficial to improve their (opto‐)electronical properties.

Herein, we design and synthesize three types of novel NHC−Au complexes and use them as starting points for Kumada polymerizations to obtain the corresponding P3HT‐NHC‐Au precursors. These precursors are then reduced to directly yield the analogous P3HT‐NHC@Au NP nanocomposites. Density functional theory (DFT) calculations and electrochromic response demonstrate that the conductive NHC linkage enables electron delocalization over the Au NP/P3HT interface. Consistently, the P3HT‐NHC@Au NP nanocomposites not only show a superior thermal and redox stability compared to PEG‐SH@Au NPs, but also an improved electrochromic response.

We targeted three different NHC‐Au‐X (X=Br, Cl) complexes, which were synthesized via multi‐step routes (Scheme 1). The monodentate initiator (**3**) is an archetypical example of an NHC ligand for coinage metals. To further increase the stability of the anchor groups, we also targeted two benzimidazoliums carrying asymmetric N‐substituents: a bipodal initiator consisting of two NHCs connected via a hexyl chain (**5**), and a variation with an SAc substituent to create an additional anchoring site (**7**). 5‐Bromo‐1*H*‐benzoimidazole (**1**) was synthesized from 4‐bromo‐2‐nitroaniline and formic acid. Alkylation of **1** with 2‐iodopropane afforded 5‐bromo‐1,3‐diisopropyl‐benzimidazolium iodide (**2**), which was converted to the corresponding Br‐NHC‐Au‐Cl complex (**3**) by the reaction of the free NHC (**2‐I**), a reactive intermediate obtained by treatment with a strong base, with Au(SMe_2_)Cl under inert conditions. 2‐Iodopropane was reacted with **1**, alkylation with dibromohexane or 4‐bromobutyl thioacetate then gives benzimidazolium salts **4** and **6**. Both Br‐DiNHC‐Au‐Br (**5**) and Br‐SAcNHC‐Au‐Br (**7**) were then obtained by reaction of **4** and **6** with Au(SMe_2_)Cl in the presence of K_2_CO_3_ (synthetic details given in the SI). All compounds were characterized by ^1^H and ^13^C NMR techniques (see SI). With the pure Br‐NHC‐Au‐X complexes in hand, chain‐growth Kumada polymerization was employed to grow P3HT from the active aryl bromide. Following a procedure by Kiriy^[12]^ and using a Ni−bipyridyl complex as intermediate catalyst, different P3HTs functionalized with NHC‐Au‐X were prepared with an initiator/monomer ratio of 1:120 (Scheme 2).

**Scheme 1:**
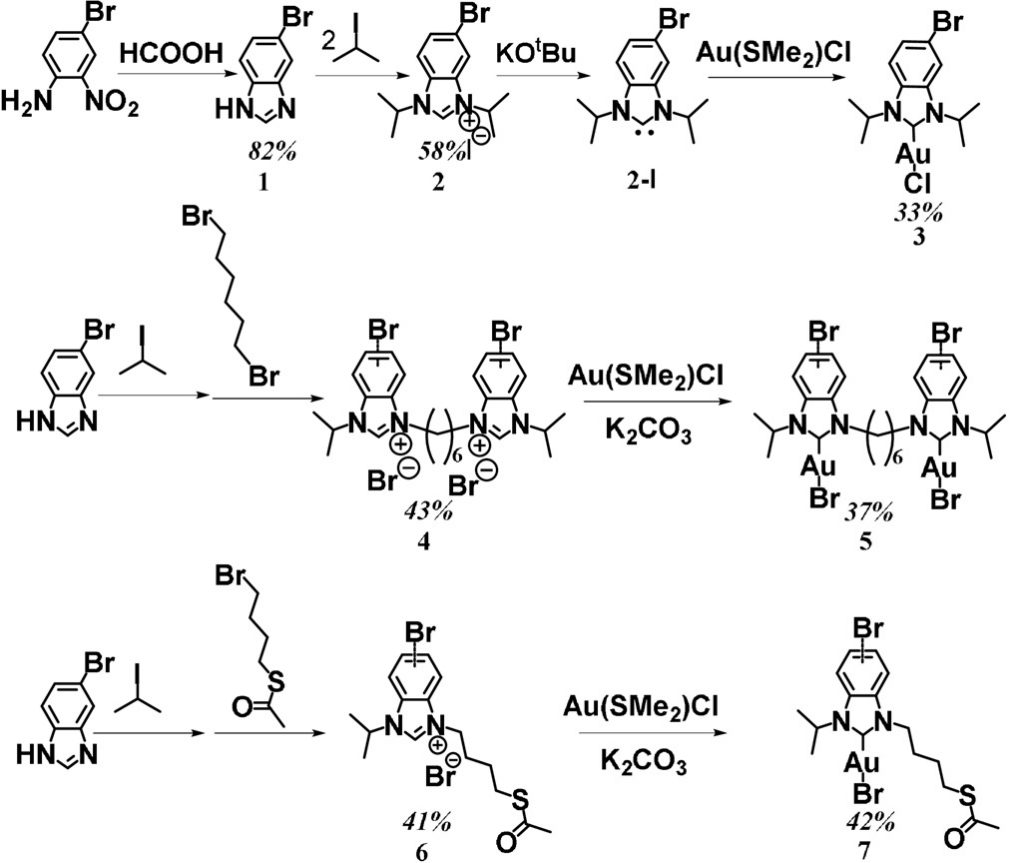
Synthetic routes for the NHC−Au complexes used as initiators. Synthetic details are given in the Supporting Information.

**Scheme 2:**
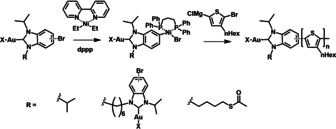
Synthesis of the P3HT‐NHC‐Au‐X polymers. X=Br for P3HT‐DiNHC‐Au and P3HT‐SAcNHC‐Au; X=Cl for P3HT‐NHC‐Au.

The polymers derived from the different NHC−Au complexes Br‐NHC‐Au‐Cl (**3**), Br‐DiNHC‐Au‐Br (**5**), and Br‐SAcNHC‐Au‐Br (**7**) are abbreviated as P3HT‐NHC‐Au, P3HT‐DiNHC‐Au, and P3HT‐SAcNHC‐Au, respectively. The number‐average molecular weights of the polymers were found to be 11 100, 9600, 12 800 g mol^−1^ (measured by GPC), respectively (Table S1), and the narrow molecular distributions (1.1–1.3) as well as the single peak at around 6.9 ppm in the ^1^H NMR spectra indicate a “head‐to‐tail” Kumada‐type polymerization. To confirm that the functional NHC−Au group is attached to the polymer chain, a polymer with lower molecular weight, P3HT‐NHC‐Au‐low, was synthesized with an initiator/monomer ratio of 1:30. In the ^1^H NMR spectrum, the signals of the NHC−Au group were shifted compared with the starting material of Br‐NHC‐Au‐Cl, suggesting the functionalization of NHC−Au (Figure S14). As the Ni catalyst can dissociate from the initiators into the reaction solution, initiating the polymerization without functional group,^[12]^ the resulting polymers are expected to contain chains without NHC−Au functionalization. This is consistent with MALDI‐TOF analysis, which shows signals relating to chains end‐functionalized with the NHC−Au group, but also non‐functionalized chains (Figure S16). As the latter do not participate in the subsequent reduction step, they can be separated in a facile way after NP formation.

We next turn to the synthesis of Au NPs from the different P3HT‐NHC‐Au^I^ precursors with NaBH_4_ as the reductant to yield P3HT‐NHC@Au NPs, P3HT‐DiNHC@Au NPs, and P3HT‐SAcNHC@Au NPs, respectively. The nanoparticles were purified by repeated centrifugation/redispersion cycles in THF and THF/CHCl_3_. As shown in Figure 2, TEM images of all Au NPs revealed small spherical particles with average diameters of 3.2, 2.5, and 6.5 nm for P3HT‐NHC@Au NPs, P3HT‐DiNHC@Au NPs, and P3HT‐SAcNHC@Au NPs.

**Figure 2:**
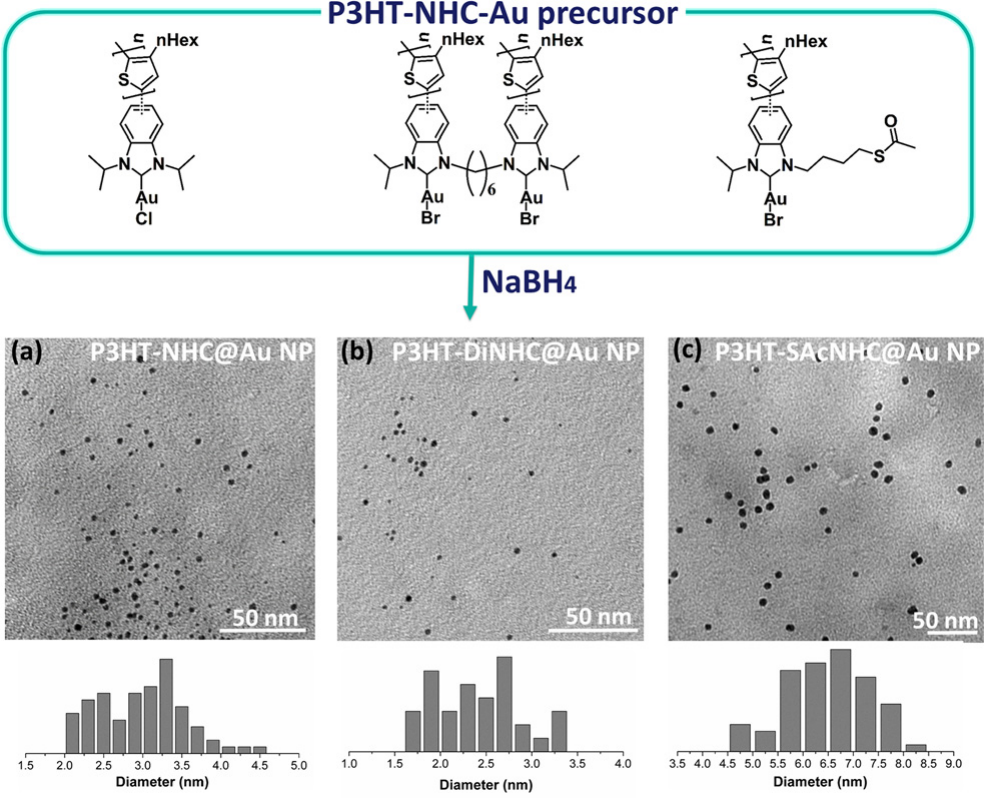
Top: molecular structures of the different P3HT‐NHC‐Au precursors; middle: TEM pictures of the corresponding P3HT‐NHC@Au NP nanocomposites derived from direct reduction with NaBH_4_; and bottom: size distributions for a) P3HT‐NHC@Au NPs (3.2±1.2 nm), b) P3HT‐DiNHC@Au NPs (2.5±0.8 nm), and c) P3HT‐SAcNHC@Au NPs (6.5±2 nm).

The Au NPs derived from P3HT‐SAcNHC‐Au showed a notably bigger average size, suggesting a beneficial influence of the close proximity of the additional thiol anchor group. The presence of the NHC on the surface of P3HT‐NHC@Au NPs, P3HT‐DiNHC@Au NPs, and P3HT‐SAcNHC@Au NPs was confirmed by X‐ray photoelectron spectroscopy (XPS), which all showed N(1s) signals at around 400 eV, consistent with the N(1s) region in the reported NHC anchors (Figures S17).

Due to the strong binding of NHCs to Au, the NHC−Au bond was reported to be extraordinary stable compared to S−Au regarding both thermal and electrochemical stability.^[4a]^ We also observed this when we compared the stability of the NHC‐based with the S‐based anchor (Figure 3).

**Figure 3:**
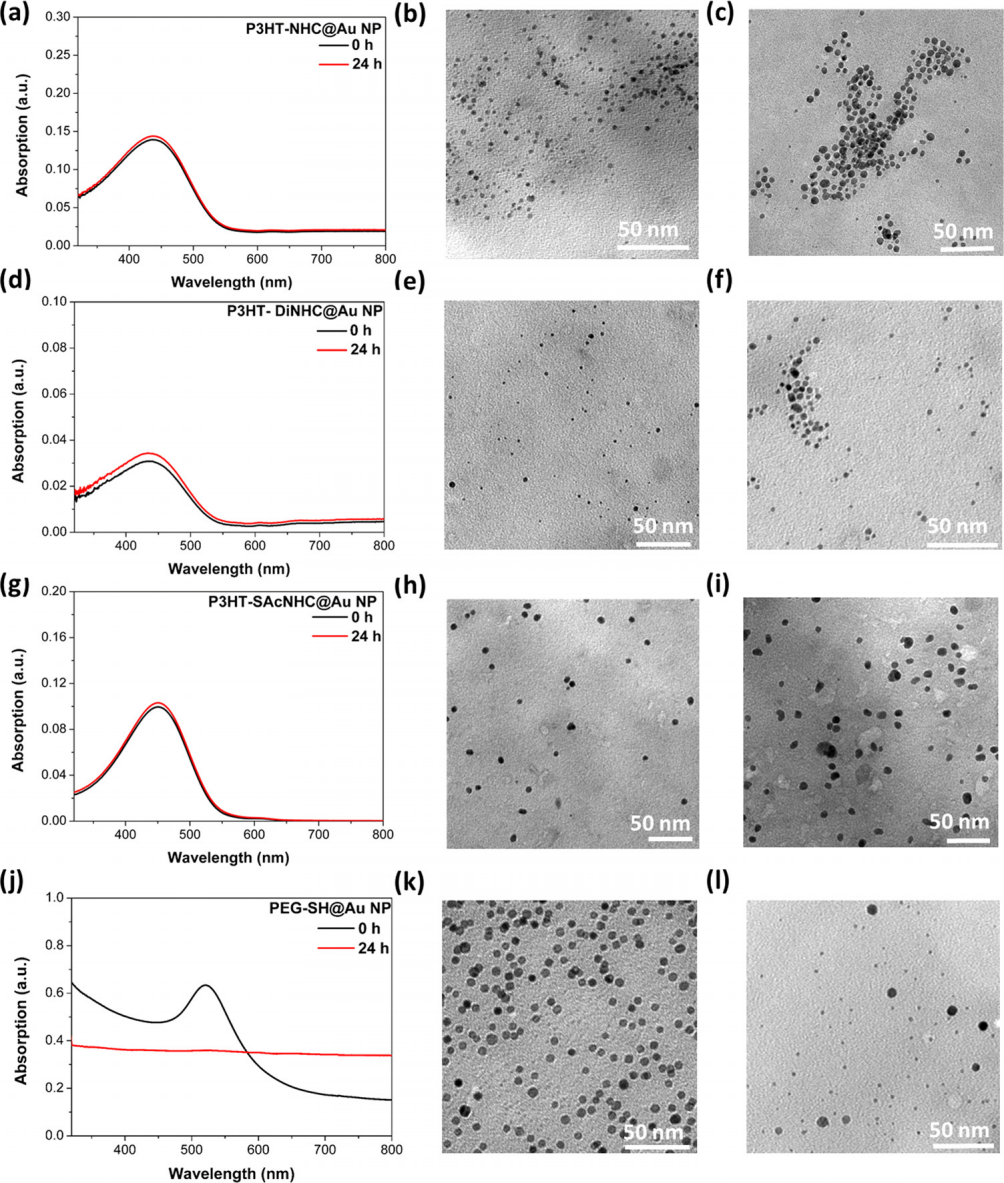
Thermal stability studies of Au NPs. Left: UV/Vis spectra of a) P3HT‐NHC@Au NPs, d) P3HT‐DiNHC@Au NPs, g) P3HT‐SAcNHC@Au NPs, and j) PEG‐SH@Au NPs, before and after heating for 24 h at 100 °C in toluene. Middle and right: TEM images of Au NPs before (middle) and after (right) heating for 24 h at 100 °C in toluene. The size of P3HT‐NHC@Au NPs changed from 3.2±1.2 to 5.6±1.6 nm (b,c). The size of P3HT‐DiNHC@Au NPs changed from 2.6±1.0 to 3.5±1.6 nm (e,f). The size of P3HT‐SAcNHC@Au NPs changed from 6.4±1.2 to 7.8±1.4 nm (h,i). The size of PEG‐SH@Au NPs changed from 5.6±0.4 to 2.1–9.6 nm (k,l).

The thermal stability of the synthesized P3HT‐NHC@Au NP composites was investigated by heating the NPs at 100 °C in toluene. Usually, the thermal stability of Au NPs is determined by monitoring the characteristic SPR band at 500–600 nm. Due to the strong absorption of the conjugated P3HT and the low intensity of the SPR band related to the small size of the NPs, the stability of the P3HT‐NHC@Au NPs could not be reliably determined by solely monitoring the SPR band, and therefore we additionally carried out TEM measurements after the thermal treatment. In these TEM images, all P3HT‐NHC@Au NPs showed a slight increase of the NP sizes, consistent with the propensity of NPs prepared from the bottom‐up method to ripen on heating.^[4b]^ Compared with P3HT‐NHC@Au NPs, both P3HT‐DiNHC@Au NPs and P3HT‐SAcNHC@Au NPs exhibited better thermal stability, which can be attributed to the increased stability of bipodal anchoring schemes. Despite the heat‐induced ripening, all NPs carrying NHC‐CP ligands show a remarkable stability up to 24 h in 100 °C toluene solutions. In comparison, NPs stabilized by sulfur‐based PEG, PEG‐SH@Au NPs, degraded under the same conditions; some NPs aggregated from 5.6 to 9.6 nm and some NPs decomposed to 2.1 nm. Also, for PEG‐SH@Au NPs, the SPR band at around 520 nm disappeared after heating (Figure 3 j).

The electrochemical properties of all P3HT‐NHC@Au NP nanocomposites were investigated by cyclic voltammetry (CV) in a three‐electrode system, with the NPs coated on ITO glass substrates as the working electrode, an Ag/AgCl as the reference electrode and a platinum wire as the counter electrode. Continuous CV scans between 0 and 0.7 V revealed only a slight decay of peak current after 50 cycles (Figure 4), indicating the good electrochemical stability of P3HT‐NHC@Au NPs, consistent with prior reports of NHC monolayers on planar gold surfaces.^[4a]^

**Figure 4:**
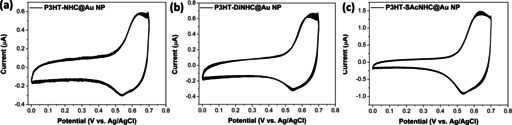
CV curves of a) P3HT‐NHC@Au NPs, b) P3HT‐DiNHC@Au NPs, and c) P3HT‐SAcNHC@Au NPs, between 0 and 0.7 V for 50 cycles at a scan rate of 50 mV s^−1^.

NHCs are highly conductive linkages, and to obtain further insight into the electronic structure of the NHC‐coupled P3HT/Au interface, density function theory (DFT) calculations (a B3LYP functional with the basis set lanl2dz effective core potential for gold and 6‐31g* for all other atoms) were performed using a simple model compound (NHC linking tetrathiophene to a single Au^0^ atom). As shown in Figure 5 a, the electron cloud is delocalized over the gold atom and the NHC in the HOMO orbit, suggesting an efficient electron transfer between Au NP and the conjugated P3HT polymer. The calculated HOMO energy level of NHC‐tetrathiophene is −4.76 eV (Figure S20), which is very close to the Fermi energy of gold (−5.1 eV).^[13]^ For Au‐NHC‐tetrathiopene, the calculated band gap decreases by 1.4 eV, from 3.1 to 1.7 eV. The decreased band gap will help the electron transfer from P3HT to Au, where the Au may work as a mediator to improve the electron transfer.

**Figure 5:**
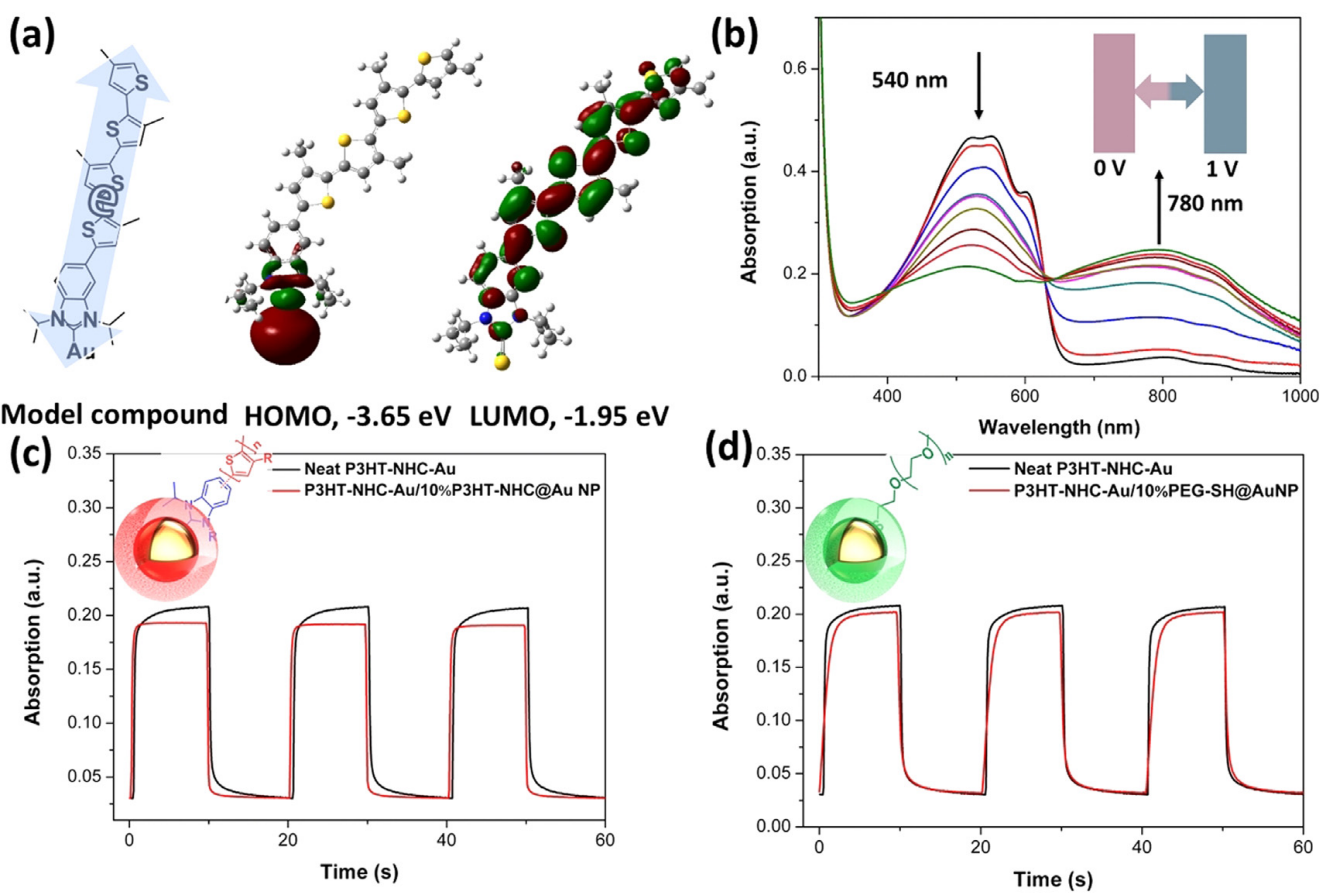
a) Molecular structure of the model compound and HOMO and LUMO distributions derived from DFT calculations. The level of theory is B3LYP/6‐31g(d,p) for H, C, and S; B3LYP/lanl2dz for Au. b) EC spectra of a P3HT‐NHC‐Au film; EC response speed of P3HT‐NHC‐Au films and c) P3HT‐NHC‐Au containing 10 % P3HT‐NHC@Au NPs, and d) P3HT‐NHC‐Au containing 10 % PEG‐SH@Au NPs.

To experimentally corroborate these findings, we carried out electrochromism (EC) experiments: P3HT is electrochromically active, and one can assume that binding the polymer to Au NPs via a conductive NHC anchor group will increase the electrochromic response speed, allowing to estimate the organic/inorganic electron transfer of P3HT‐NHC@Au NPs. To obtain good films on ITO glass substrates, 10 % P3HT‐NHC@Au NP nanocomposites were blended into the neat corresponding polymer. Spectroelectrochemical analyses were carried out to evaluate the electrochromic properties of neat P3HT‐NHC‐Au and the composite containing 10 % P3HT‐NHC@Au NPs. The changes observed in the electrochromic spectra are similar for both films: upon increasing the applied potential from 0 to 1 V, the polymer film changed color from red to light blue. While the neutral absorption maximum at 540 nm decreased, a new broad absorption band centered at 780 nm appeared. The response speed was studied by monitoring the absorption changes at 780 nm under applied square wave potential between 0 and 1 V. The response time, calculated at 90 % of full absorption change, was found to be 1.21/1.05 s for neat P3HT‐NHC‐Au and 0.79/0.52 s for the composite containing 10 % P3HT‐NHC@Au NPs. On the other hand, when blending with 10 % PEG‐SH@Au NPs into P3HT‐NHC‐Au, the resulting response speed decreased significantly to 1.31/1.06 s. The increase in response speed upon adding P3HT‐NHC@Au NPs indicates that the P3HT‐NHC@Au NP systems promote the electron transfer in the electrochromic P3HT films.

In summary, we present a synthetic pathway to (i) use functionalized NHC‐Au‐X complexes as initiators for Kumada‐type polymerizations to yield monofunctionalized regioregular P3HTs with narrow weight distributions; and (ii) to obtain the corresponding P3HT‐NHC@Au NP systems as well‐dispersed NPs via direct reduction. In comparison with PEG‐stabilized Au NPs, the synthesized P3HT‐NHC@Au NP systems show excellent thermal and electrochemical redox stabilities. DFT calculations on model compounds indicate electron delocalization across the organic/inorganic interface via the NHC anchor group, consistent with the experimentally determined higher electrochromic response speed of the nanocomposites: Blending 10 % of P3HT‐NHC@Au NPs into a P3HT‐NHC‐Au film improves the responsive speed significantly, from 1.21/1.05 s for neat P3HT‐NHC‐Au to 0.79/0.52 s for the blend. In comparison, a blend containing 10 % PEG‐stabilized Au NPs showed a decrease in response speed to 1.31/1.06 s. Linking Au NPs and conjugated CPs with stable and conductive NHC anchor groups opens up exciting opportunities for developing a variety of new electronically coupled organic–inorganic nanocomposites for (opto‐)electronic applications. To study the effect of electron delocalization across the organic/inorganic interface of optoelectronic assemblies, we currently work on realizing CP‐NHC@Au NP systems with larger NP sizes.

## Conflict of interest

The authors declare no conflict of interest.

## Supporting information

As a service to our authors and readers, this journal provides supporting information supplied by the authors. Such materials are peer reviewed and may be re‐organized for online delivery, but are not copy‐edited or typeset. Technical support issues arising from supporting information (other than missing files) should be addressed to the authors.

Supplementary
