# Supplementary material for: Poly(3‐hexylthiophene)s Functionalized with N‐Heterocyclic Carbenes as Robust and Conductive Ligands for the Stabilization of Gold Nanoparticles
Source: Angew Chem Int Ed Engl. 2020 Dec 29;60(8):3912–7. doi: 10.1002/anie.202012216 (PMC7898828; doi:10.1002/anie.202012216)
Supplement: Supplementary file 1 — Supplementary [file ANIE-60-3912-s001.pdf]

Angewandte Chemie
Eine Zeitschrift der Gesellschaft Deutscher Chemiker

Supporting Information

Poly(3-hexylthiophene)s Functionalized with N-Heterocyclic Carbenes as Robust and Conductive Ligands for the Stabilization of Gold Nanoparticles

Ningwei Sun, Shi-Tong Zhang, Frank Simon, Anja Maria Steiner, Jonas Schubert, Yixuan Du, Zhi Qiao, Andreas Fery, and Franziska Lissel**

anie_202012216_sm_miscellaneous_information.pdf

Table of Contents

Table of Contents S2
(1) Experimental Methods and Instrumentation S3
Nuclear Magnetic Resonance (NMR) Spectroscopy S3
Gel Permeation Chromatography (GPC) S3
Ultraviolet-visible (UV/Vis) Spectroscopy S3
Cyclic voltammetry (CV) and Electrochromism (EC) S3
Transmission Electron Microscopy (TEM) S3
X-ray Photoelectron Spectroscopy (XPS) S3
MALDI-TOF mass spectra S4
Density function theory (DFT) S4
(2) Synthesis S5
Materials S5
Synthesis of monomers S5
Synthesis of 5-bromo-1*H*-benzoimidazole (1): S5
Synthesis of 5-bromo-1,3-diisopropyl-benzimidazolium iodide (2): S5
Synthesis of (5-bromo-1,3-diisopropyl-benzimidazole)gold chloride (Br-NHC-Au-Cl, 3): S5
Synthesis of 1,6-di(5/6-bromo-1–isopropyl-benzimidazolium bromide)-hexane (4) S6
Synthesis of 1,6-(di(5/6-bromo-1–isopropyl-benzimidazole)gold bromide)-hexane (2Br-NHC-Au-Br, 5): S6
Synthesis of 5/6-bromo-1-isopropyl-3-3-(acetylthio)butyl)-benzimidazolium bromide (6): S7
(5/6-bromo-1-isopropyl-3-3-(acetylthio)butyl)-benzimidazole)gold bromide (Br-SAcNHC-AuBr, 7):7
Synthesis of NHC-Au-functionalized P3HTs based on Kumada polymerization S8
Synthesis of NHC-Au-based initiators: S8
Polymerization of NHC-Au-functionalized P3HT: S8
Endcapping efficiency: S8
Synthesis of Au NPs derived from NHC-Au-functionalized P3HT S9
Synthesis of PEG-SH@Au NP S9
Synthesis of CtaC@Au NP S9
Synthesis of PEG-SH@Au NP S9
(3) Experimental Data S10
Nuclear Magnetic Resonance (NMR) Spectroscopy S10
Gel Permeation Chromatography (GPC) S17
MALDI-TOF S18
X-ray Photoelectron Spectroscopy (XPS) S19
DFT S19
References S21

(1) Experimental Methods and Instrumentation

Nuclear Magnetic Resonance (NMR) Spectroscopy

A Bruker Avance III 500 spectrometer was used to record ^{1}H NMR spectra at 500 MHz and ^{13}C NMR spectra at 126 MHz. The spectra were referenced to the residual solvent signals ($CDCl_3$: δ(^{1}H) = 7.26 ppm, DMSO-d_6: δ(^{1}H) = 2.50 ppm). The following abbreviations were used for ^{1}H NMR spectra data as listed: s - singlet, d - doublet, dd - doublet of doublet, t – triplet and m -multiplet.

Gel Permeation Chromatography (GPC)

GPC measurements were performed against polystyrene (PS) standards using an Infinity 1260 with equipped with RI and UV/Vis detection. Chloroform was used as eluent at 40 °C and the GPC set to a 1 mL/min flow rate. Polymer samples were dissolved (2.5-3.0 mg/mL) prior to the measurements.

Ultraviolet-visible (UV/Vis) Spectroscopy

UV-vis spectra were measured on UV/vis/NIR spectrophotometer Cary 5000 (Agilent Technologies Deutschland GmbH).

Cyclic voltammetry (CV) and Electrochromism (EC)

Electrochemical analyses were carried out on Autolab PGSTAT302N workstation. Cyclic voltammetry (CV) measurements were carried out in a 3-electrode setup with platinum counter electrode and Ag/AgCl reference electrode. NPs coated on ITO substrates were used as working electrodes. 0.1 M tetrabutylammonium hexafluorophosphate acetonitrile solution was used as the electrolyte. Spectroelectrochemical measurements were carried out by coupling the electrochemistry workstation with the UV-vis spectrophotometer. The setup was similar with CV measurements; the polymer solutions or solutions of polymers blended with NPs were spin-coated on ITO substrates (~10 mg/mL of polymer solution in chloroform).

Transmission Electron Microscopy (TEM)

TEM images were obtained using a Zeiss Libra 120 with an accelerating voltage of 120 kV. Samples were prepared by placing a 2 µL droplet of the diluted NP dispersions on TEM grids (Cu, 200 Mesh, coated with carbon film; Science Services GmbH).

X-ray Photoelectron Spectroscopy (XPS)

The X-ray Photoelectron Spectroscopy (XPS) studies were carried out using of an AXIS ULTRA photoelectron spectrometer (KRATOS ANALYTICAL, Manchester, England). The spectrometer was equipped with a monochromatic Al Kα (hν = 1486.6 eV) X-ray source of 300 W at 15 kV. The kinetic energy of the photoelectrons was determined with a hemispherical analyzer set to a pass energy of 160 eV for wide-scan spectra and 20 eV for high-resolution spectra. During all measurements electrostatic charging of the sample was compensated by means of a low energy electron source working in combination with a magnetic immersion lens. Later, all recorded peaks were shifted by the same value that was necessary to set the C 1s peak to 285.00 eV.
Quantitative elemental compositions were determined from peak areas using experimentally determined sensitivity factors and the spectrometer transmission function. Spectrum background was subtracted according to Shirley. The high-resolution spectra were

deconvoluted by means of the Kratos spectra deconvolution software. Free parameters of component peaks were their binding energy (BE), height, full width at half maximum and the Gaussian-Lorentzian ratio.

MALDI-TOF mass spectra

All MALDI spectra were recorded with a “Bruker autoflex speed” MALDI-TOF/TOF in reflectron positive mode. The laser of this instrument is a smartbeam-II with a wavelength of 355 nm. The software for measuring the spectra was flexControl 3.4 from Bruker. All measurements were carried out using a standard set of parameters. Samples for measurements were prepared in $CHCl_3$ by mixing 5 µL of polymer solution with 50 µL of matrix (*trans*-2-[3-(4- tert-Butylphenyl)-2-methyl-2-propenylidene]malononitrile) solution. A total of 1 µL of this mixture was deposited on the plate, and after evaporation of the solvent, measurements were performed in reflectron mode.

Density function theory (DFT)

All DFT calculations were carried out with the Gaussian 09 B.01 Package with the basis set LANL2DZ+ effective core potential for gold and 6-31g* for all other atoms.

(2) Synthesis

Materials

Unless noted otherwise, all chemicals were obtained from commercial suppliers and used without further treatment. Diethyldipyridylnickel ($Et_2Ni(bipy)$) was synthesized according to a reported literature.[S1]

Synthesis of monomers

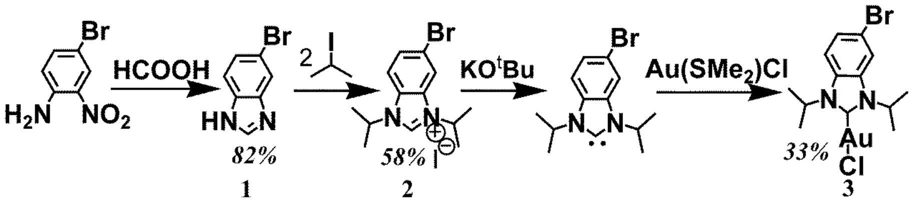

Scheme S1 Synthesis of Br-NHC-Au-Cl (**3**).

Synthesis of 5-bromo-1*H*-benzoimidazole (1):

5-Bromo-1H-benzoimidazole was synthesized by modifying a literature protocol [S2]: 5-Bromo-2-nitroaniline (10 g, 46 mmol), NH_4Cl (24.6 g, 460 mmol) and Fe powder (25.7 g, 460 mmol) were added into a 2-neck flask, which was then evacuated, heated and flooded with argon for three times. In a separate flask, formic acid (175 mL) and isopropanol (250 mL) were purged with argon for 20 min, and then was transferred into the first flask. The resulting mixture was heated at 80 °C for 3 h under argon atmosphere. After cooling, the mixture was filtered through diatomite and washed with isopropanol (3 times, 60 mL each). The filtrate was evaporated to dryness and aqueous $NaHCO_3$ solution was added to adjust the pH to 7. Then, chloroform (3 portions, 60 mL each) was used to extract the mixture, and the combined chloroform phase was dried over anhydrous $MgSO_4$. The solvent was removed *via* rotary evaporation, and the crude product was purified by recrystallization from ethanol/diethyl ether to yield the target product (7.2 g, yield: 82%).

^1H NMR (500 MHz, DMSO-d_6) 12.58 (s, 1H), 8.25 (s, 1H), 7.80 (s, 1H), 7.56 (s, 1H), 7.33 (d, J = 8.4 Hz, 1H).

Synthesis of 5-bromo-1,3-diisopropyl-benzimidazolium iodide (2):

5-Bromo-1,3-diisopropyl-benzimidazolium iodide was synthesized by modifying a literature protocol[S3]: A mixture of 5-bromo-1*H*-benzoimidazole (**1**) (5 g, 25 mmol), K_2CO_3 (5.85 g, 30 mmol), 2-iodopropane (8.7 g, 50 mmol) and acetonitrile (100 mL) was stirred at 85 °C for 12 h. Then another 34.8 g of 2-iodopropane was added into the mixture, and stirring was continued at 85 °C for 60 h. After cooling to room temperature, the mixture was evaporated to remove unreacted 2-iodopropane and solvent. Dichloromethane (DCM) (300 mL) was added to the residue and the suspension was filtered over diatomite. After removing the solvent, the crude solid was purified by recrystallization from methanol/diethyl ether to give white crystals (6.26 g, yield: 58 %).

^1H NMR (500 MHz, DMSO-d_6): δ 9.79 (s, 1H), 8.51 (d, J = 1.8 Hz, 1H), 8.13 (d, J = 8.8 Hz, 1H), 7.87 (dd, J = 8.9, 1.8 Hz, 1H), 5.16 – 4.99 (m, 2H), 1.63 (dd, J = 6.7, 3.4 Hz, 12H).

Synthesis of (5-bromo-1,3-diisopropyl-benzimidazole)gold chloride (Br-NHC-Au-Cl, 3):

In a glovebox, 5-bromo-1,3-diisopropyl-benzimidazolium iodide (**2**) (1 g, 2.45 mmol) was suspended in anhydrous THF (60 mL). KO^{t}Bu (0.3 g, 2.69 mmol) was added to the suspension and the reaction was stirred for 1 h. The mixture was then filtered through diatomite, Au(SMe_2)Cl (0.75 g, 2.56 mmol) was added to the filtrate and the reaction was stirred for 6 h protected from light. Activated carbon (~0.5 mg) was added to the mixture, which was stirred overnight. The mixture was filtered through diatomite, and the filtrate

was evaporated to remove all solvent. The crude product was purified by column chromatography (DCM/*n*-hexanes 1:4) to give the target product (0.41 g, yield: 33%).

^{1}H NMR (500 MHz, Chloroform-*d*): δ 7.72 (d, *J* = 1.6 Hz, 1H), 7.44 (d, *J* = 8.7 Hz, 1H), 7.41 (dd, *J* = 8.8, 1.6 Hz, 1H), 5.54 – 5.30 (m, 2H), 1.65 (dd, *J* = 7.0, 5.0 Hz, 12H).

^{13}C NMR (126 MHz, Chloroform-*d*): δ 187.23, 133.41, 131.27, 127.24, 117.48, 116.04, 114.11, 53.99, 21.82.

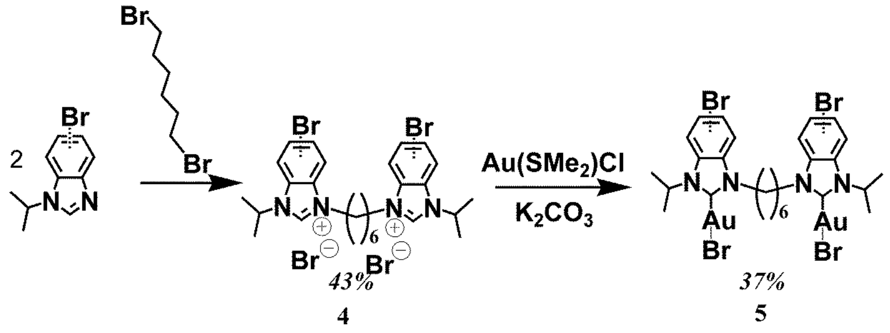

Scheme S2 Synthesis Br-DiNHC-Au-Br (**5**)

Synthesis of 1,6-di(5/6-bromo-1–isopropyl-benzimidazolium bromide)-hexane (4)

A mixture of 5-bromo-1*H*-benzoimidazole (**1**) (0.33 g, 1.67 mmol), Cs_2CO_3 (0.65 g, 2.0 mmol), 2-iodopropane (0.31 g, 1.8 mmol) and acetonitrile (10 mL) was stirred at 85 °C for 12 h. Then the mixture was filtered hot to remove insoluble inorganic salts, and the filtrate was dried by rotary evaporator to remove all volatiles. The oily residual was transferred into a high pressure tube with 2 ml of acetonitrile. After adding 1,6-dibromohexane (0.2 g, 0.82 mmol), the high pressure was sealed and heated at 110 °C for 48 h. After cooling down, a dark oily solid precipitated. The supernatant solution was carefully poured out and the dark oily solid was purified by precipitating a DCM solution into *n*-hexanes three times. Subsequent drying under vacuum gave a sticky solid (0.31 g, yield: 47%). The product is a (roughly) 1:1 mixture of two isomers (5- and 6-substitution) that are difficult to separate by column chromatography.

^{1}H NMR (500 MHz, DMSO -*d*$_6$) δ 10.18 – 9.92 (m, 1H), 8.58 – 8.45 (m, 1H), 8.19 – 8.05 (m, 1H), 7.92 – 7.83 (m, 1H), 5.07 (m, 1H), 4.56 – 4.36 (m, 2H), 2.00 – 1.87 (m, 2H), 1.71 – 1.55 (m, 6H), 1.49 – 1.31 (m, 2H).

Synthesis of 1,6-(di(5/6-bromo-1–isopropyl-benzimidazole)gold bromide)-hexane (2Br-NHC-Au-Br, 5):

1,6-Di(5/6-bromo-1–isopropyl-benzimidazolium bromide)-hexane (**4**) (0.157 g, 0.2 mmol), K_2CO_3 (0.138 g, 1 mmol) and Au(SMe_2)Cl (0.118 g, 0.4 mmol) and acetone (10mL) were added into a flask. The mixture was stirred at 65 °C for 10 h. After cooling, the solvent was evaporated under vacuum and the residue was dissolved in DCM. Activated carbon (~0.1 mg) was added to the mixture, which was stirred for 4 h. The mixture was then filtered through diatomite and the solvent was removed by evaporation.

A further purification was performed by column chromatography (DCM/*n*-hexanes, gradients from 1:6 to 1:4) to give the target product (96 mg, yield: 37%) as a mixture of two isomers (5- and 6-substitution).

^{1}H NMR (500 MHz, Chloroform-*d*): δ 7.74 – 7.62 (m, 1H), 7.53 – 7.39 (m, 2H), 5.41 – 5.27 (m, 1H), 4.48 – 4.34 (m, 2H), 1.98 – 1.85 (m, 2H), 1.74 – 1.63 (m, 6H), 1.54 – 1.44 (m, 2H).

^{13}C NMR (126 MHz, Chloroform-*d*): δ 181.45, 181.39, 134.70, 132.71, 130.69, 127.86, 127.85, 127.45, 127.41, 118.00, 115.74, 115.71, 115.02, 115.00, 113.91, 113.89, 113.36, 113.30, 77.27, 77.02, 76.76, 54.19, 54.11, 48.58, 48.49, 48.39, 29.67, 29.53, 29.48, 29.34, 25.73, 25.72, 25.67, 25.65, 21.88.

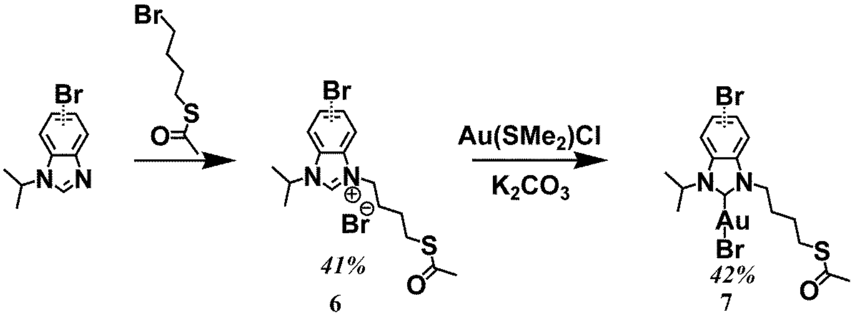

Scheme 3 Synthesis of Br-SAcNHC-Au-Br (**7**).

Synthesis of 5/6-bromo-1-isopropyl-3-3-(acetylthio)butyl)-benzimidazolium bromide (6):

A mixture of 5-bromo-1*H*-benzoimidazole (**1**) (0.33 g, 1.67 mmol), Cs_2CO_3 (0.65 g, 2.0 mmol), 2-iodopropane (0.31 g, 1.8 mmol) and acetonitrile (10 mL) was stirred at 85 °C for 12 h. Then the mixture was filtered hot to remove all insoluble inorganic salts, and the filtrate was dried by rotary evaporator to remove all volatiles. The oily residual was transferred into a high pressure tube with 3 ml acetonitrile. After adding 1,6-dibromohexane (0.425 g, 2.0 mmol), the high pressure was sealed and heated at 100 °C for 48 h. After cooling down, a dark oily solid precipitated. The supernatant solution was carefully poured out and the dark oily solid was purified by precipitating a DCM solution into *n*-hexanes three times. Subsequent drying under vacuum gave a sticky solid (0.36 g, yield: 4%). The product is a (roughly) 1:1 mixture of two isomers (5- and 6-substitution) that are difficult to separate by column chromatography.

^{1}H NMR (500 MHz, DMSO -d_6) δ 9.96 – 9.85 (m, 1H), 8.58 – 8.47 (m, 1H), 8.18 – 8.06 (m, 1H), 7.96 – 7.80 (m, 1H), 5.16 – 4.98 (m, 1H), 4.56 – 4.40 (m, 2H), 3.68 – 3.59 (m, 2H), 2.99 – 2.85 (m, 2H), 2.37 – 2.28 (m, 3H), 2.03 – 1.92 (m, 2H), 1.83 – 1.72 (m, 2H), 1.64 – 1.59 (m, 6H), 1.59 – 1.55 (m, 2H).

(5/6-bromo-1-isopropyl-3-3-(acetylthio)butyl)-benzimidazole)gold bromide (Br-SAcNHC-AuBr, 7):

5/6-Bromo-1-isopropyl-3-3-(acetylthio)butyl)-benzimidazolium bromide (**6**) (0.144 g, 0.3 mmol), K_2CO_3 (0.21 g, 1.5 mmol) and $Au(SMe_2)Cl$ (0.088g, 0.3 mmol) and acetone (12 mL) were added into a flask. The mixture was stirred at 65 °C for 8 h. After cooling, the solvent was evaporated under vacuum and the residue was dissolved in DCM. Activated carbon (~0.1 mg) was added to the mixture, which was stirred for 4 h. The mixture was then filtered through diatomite and the solvent was removed by evaporation. A further purification was performed by column chromatography (DCM/*n*-hexanes, gradients from 1:5 to 1:2) to give the target product (84 mg, yield: 42%) as a mixture of two isomers (5- and 6-substitution).

^{1}H NMR (500 MHz, Chloroform-*d*): δ 7.72 (s, 1H), 7.59 (s, 1H), 7.51 – 7.42 (m, 3H), 7.38 – 7.28 (m, 1H), 5.42 – 5.30 (m, 2H), 4.47 – 4.35 (m, 4H), 2.92 – 2.80 (m, 4H), 2.29 – 2.20 (m, 6H), 1.99 – 1.88 (m, 4H), 1.71 – 1.56 (m, 16H).

^{13}C NMR (126 MHz, Chloroform-*d*): δ195.67, 195.62, 181.65, 181.58, 134.69, 132.76, 132.64, 130.69, 127.74, 127.44, 117.96, 117.73, 115.83, 114.77, 114.02, 112.91, 77.34, 77.09, 76.83, 54.21, 54.12, 48.56, 48.49, 30.69, 30.67, 28.69, 28.53, 28.26, 28.21, 26.77, 26.71, 21.86, 21.81.

Synthesis of NHC-Au-functionalized P3HTs based on Kumada polymerization

Synthesis of NHC-Au-based initiators:

The initiators based on NHC-Au complexes were synthesized by modifying a protocol developed by Kiriy et al [S4] that includes the initial reaction of arylhalides with Et_2Ni(bipy), followed by the addition of diphenylphosphinopropane (dppp) ligand.
The polymerization using Br-NHC-Au-Cl is presented as an example:

In a glovebox, Et_2Ni(bipy) (43.5 mg, 0.16 mmol) in 2 mL of anhydrous THF was added dropwise to Br-NHC-Au-Cl (**3**) (106 mg, 0.2 mmol) in 2 mL of anhydrous THF. After stirring at room temperature for 3 h, the green color inherent to Et_2Ni(bipy) changed to red, indicating the formation of the corresponding Ni(bipy)-Br complex. Then, 86.5 mg (2.1 mmol) of dppp in 2 mL THF was added slowly to the above solution and the reaction mixture was stirred until the color changed from red to yellow. The product was used for the Kumada-type polymerization without any further purification.

Polymerization of NHC-Au-functionalized P3HT:

A 25 mL flask was purged by argon three times, and 2-bromo-3-hexyl-5-iodothiphene (0.373 g, 1 mmol) in anhydrous THF (10 mL) was added. The resulting solution was cooled to 0 °C, and *i*PrMgCl (2M in THF, 0.48 mL) was added. After stirring at 0 °C for 1 h, the resulting yellow solution was added to the initiator solution (see above) with an initiator/monomer ratio of 1/120. A color change from yellow to deep red was observed within a few minutes, indicating the forming of P3HT. After reacting for 60 min, the polymerization was quenched by adding 5 M HCl in methanol. The polymer was precipitated in methanol and the methanol solution filtered off. The crude polymer was further purified by subsequently washing with methanol, acetone and *n*-hexanes several times. Finally, the polymer was dissolved in a small amount of chloroform and precipitated in methanol to afford a dark purple solid.

Endcapping efficiency:

From the average number molecular weights measured by GPC, the average number of repeat units of P3HT-NHC-Au, P3HT-DiNHC-Au and P3HT-SAcNHC-Au were estimated to be 64, 52, and 73, respectively. The corresponding monomer conversions were 53%, 43%, and 60%, respectively. However, the Ni catalyst is known to dissociate from the initiators into the reaction solution during the polymerization process, generating polymer chains which do not carry the aryl group of the original iniator. In our case, the initiators contain a gold atom, which may make the polymerization more complicated. The molecular weights determined from GPC and NMR did not match well. To confirm the attachment of the NHC-Au group on the P3HT chains, a polymer with a lower molecular weight (P3HT-NHC-Au-low), was synthesized at the initiator/monomer ratio of 1/30.
P3HT-NHC-Au-low is expected to allow a more accurate integration of the NMR peaks. For this polymer, the number-average molecular weight was determined to be 2800 according to GPC, while the molecular weight calculated from ^{1}H NMR spectrum was 5900. As some polymer chains do not carry the functional group, the integration in ^{1}H NMR will overestimate the ration repeating unit/endgroup, and consequently overestimate the molecular weight. This is also consistent with the MALDI-TOF data (see below, Fig. S16, Table S2), which also indicate that only about 50%& of the polymer chains carry the NHC-Au group. But even if the functionalization yield is not high, the non-functionalized polymers can be removed in a facile way after the NP formation: Only polymer chains carrying an NHC-Au group will participate in the reduction to form the nanoparticles, the unfunctionalized free polymer chains can then be removed via repeated wash/centrifugation steps.

Synthesis of Au NPs derived from NHC-Au-functionalized P3HT

To a stirring solution of NHC-Au-functionalized P3HT (20 mg) in 5 mL of chloroform/THF (4:1), was quickly added a freshly prepared solution of $NaBH_4$ (2 mg in 0.5 mL ethanol). The solution was stirred for 24 h, then filtered through a cotton wool and the filtrate was evaporated to remove the solvents. The residue was washed with water (3*10 mL) and methanol (3*10 mL). The crude NPs were further purified by several centrifugation/redispersion cycles using THF and chloroform/THF (4:1).

Synthesis of PEG-SH@Au NP

Synthesis of CtaC@Au NP

First, Au NPs with hexadecyltrimethylammonium chloride (CtaC) as the ligand were synthesized by following a modified literature report [S5]: Aqueous solutions hexadecyltrimethylammonium bromide (CtaB, 100 mM, 9.4 mL) and $HAuCl_4$ (2.5 mM, 18.4 μL) were mixed and stored at 35 °C for 10 min. Then, 600 μL of a freshly prepared $NaBH_4$ (10 mM) solution was added quickly under vigorous stirring. Stirring was stopped after 30 s, and the seeds were then stored for 25 min at 40 °C to completely remove excess $NaBH_4$.
Second, aqueous solutions of CtaC (200 mM, 40 mL), ascorbic acid (AA) (1 M, 6 mL), and the initial CtaB-capped Au clusters (5 mL) were mixed in a 100 mL beaker. An aqueous $HAuCl_4$ solution (1.0 mM, 40 mL) was then quickly injected under stirring. The reaction was allowed to continue at room temperature for 15 min. The target Au NPs (5.6 nm±0.4 nm measured by TEM) were collected by centrifugation twice in aqueous CtaC solution (20 mM).

Synthesis of PEG-SH@Au NP

With the CtaC@Au NPs in hand, a ligand exchange was carried out to obtain PEG-SH@Au NP: PEG-SH (molecular weight: 6000, 0.1 mg/mL in $CHCl_3$, 0.4 mL) and CtaC@Au NP (1 mg/mL in water, 2 mL) were mixed into a vial with shaking. Then $CHCl_3$ (1 mL) and methanol (4 mL) were added subsequently to promote the phase separation. After storing for 1 h, the PEG-SH@Au NP in $CHCl_3$ were collected.

(3) Experimental Data

Nuclear Magnetic Resonance (NMR) Spectroscopy

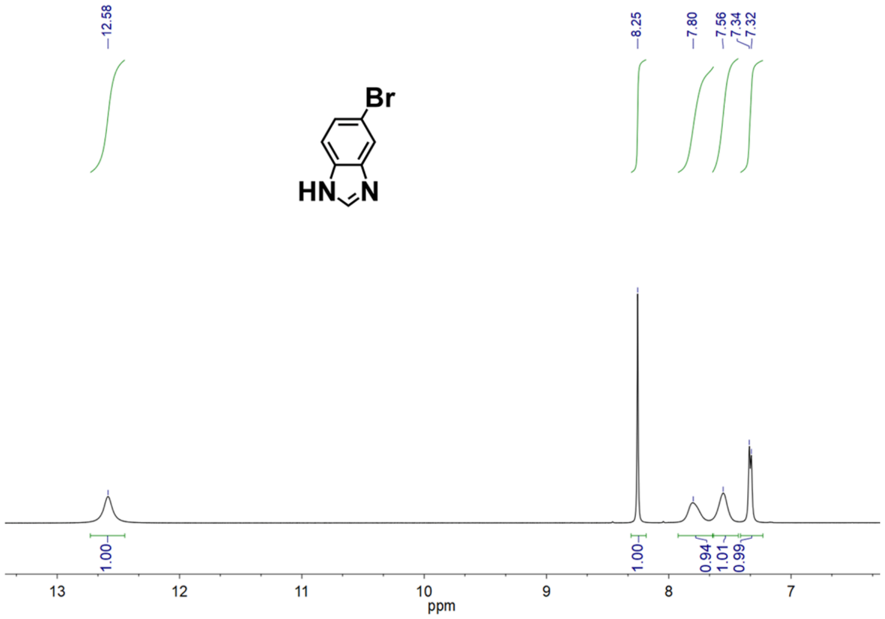

Figure S1. ^{1}H NMR spectrum of (**1**) in DMSO-d_6.

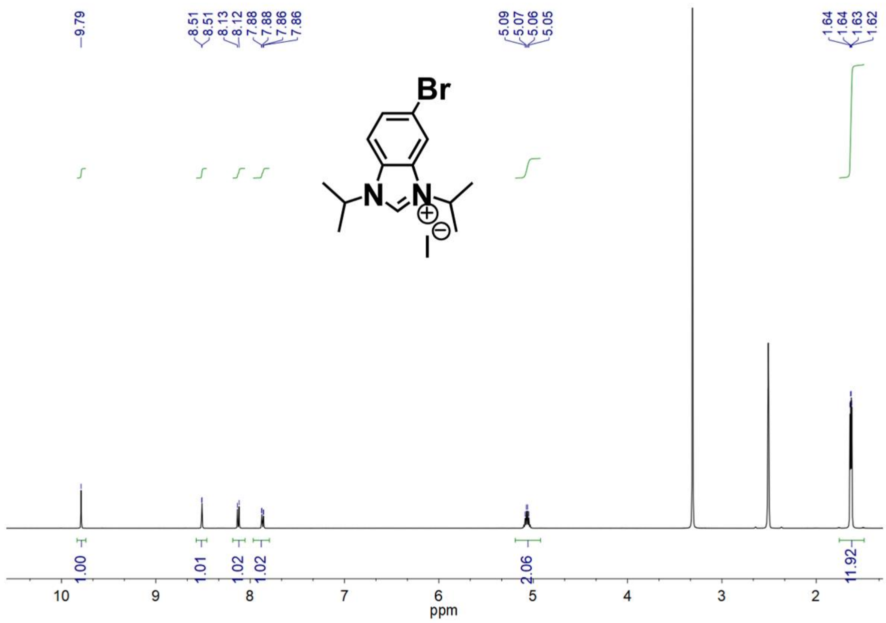

Figure S2. ^{1}H NMR spectrum of (**2**) in DMSO-d_6.

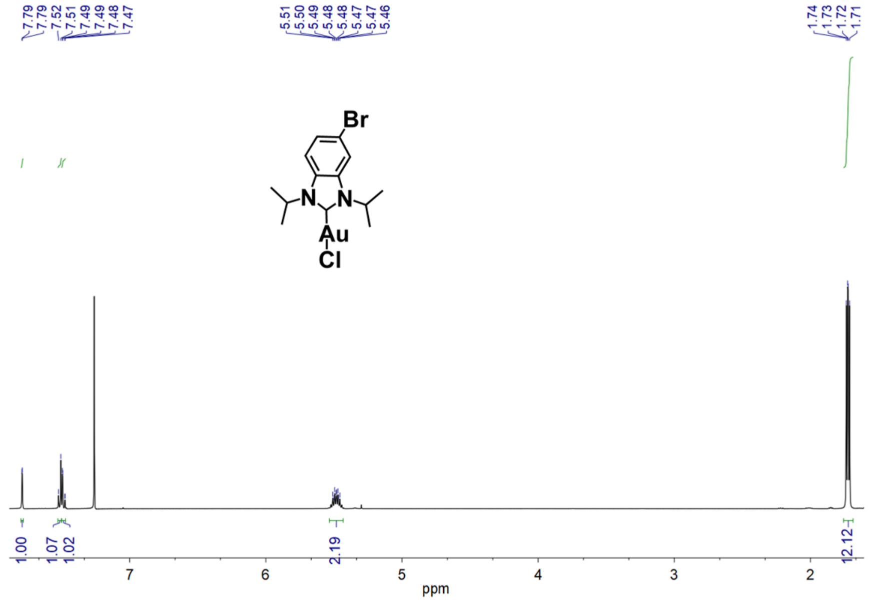

Figure S3. ^{1}H NMR spectrum of (**3**) in $CDCl_3$.

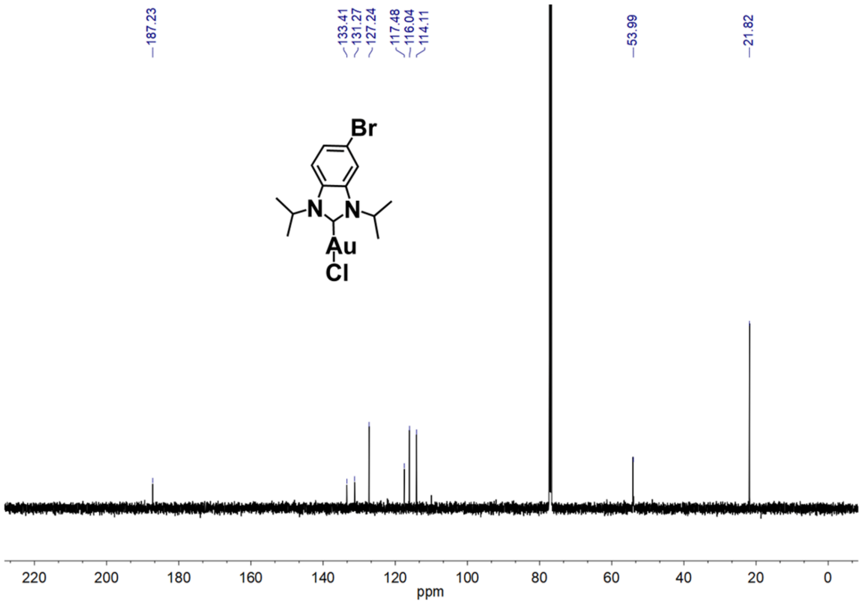

Figure S4. ^{13}C NMR spectrum of (**3**) in $CDCl_3$.

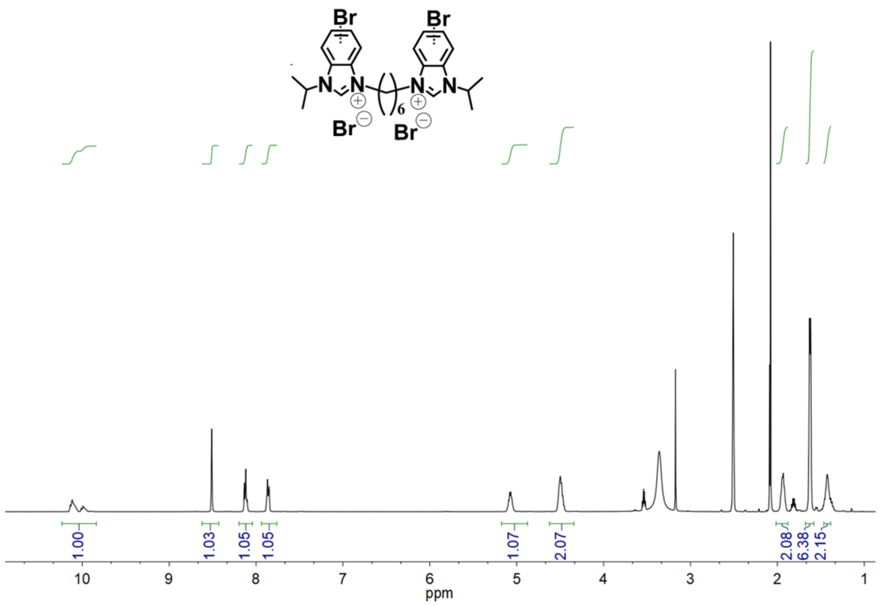

Figure S5. ^{1}H NMR spectrum of (**4**) DMSO-*d*$_6$.

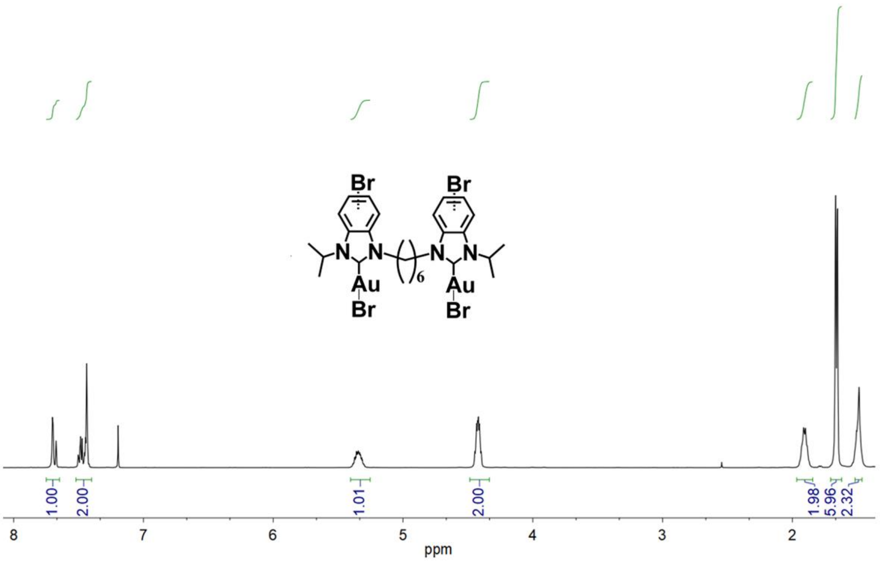

Figure S6. ^{1}H NMR spectrum of (**5**) in $CDCl_3$.

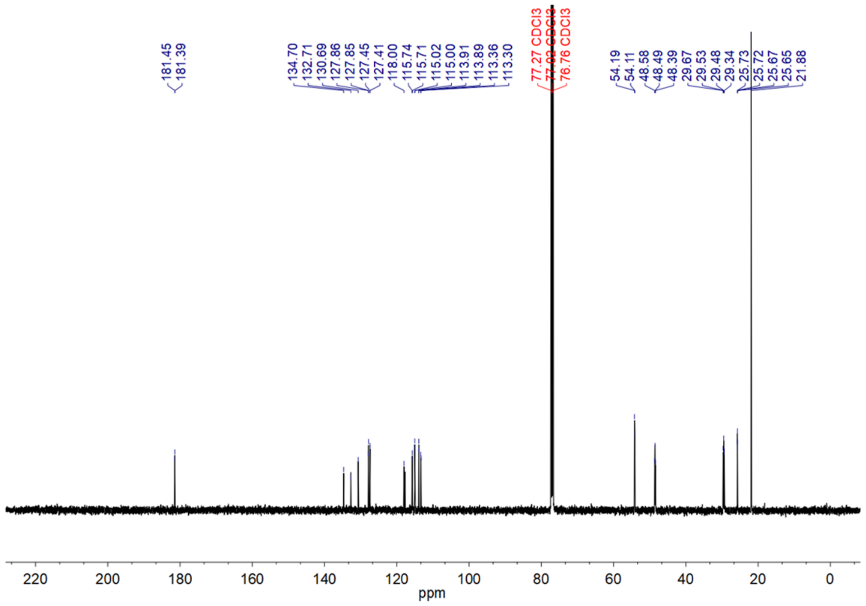

Figure S7. ^{13}C NMR spectrum of (**5**) in $CDCl_3$.

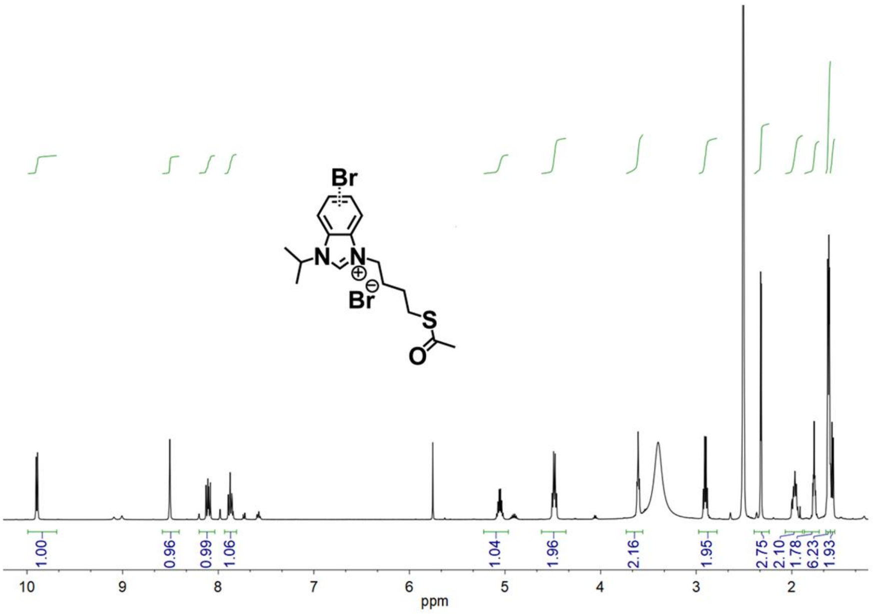

Figure S8. ^{1}H NMR spectrum of (**6**) DMSO-d_6.

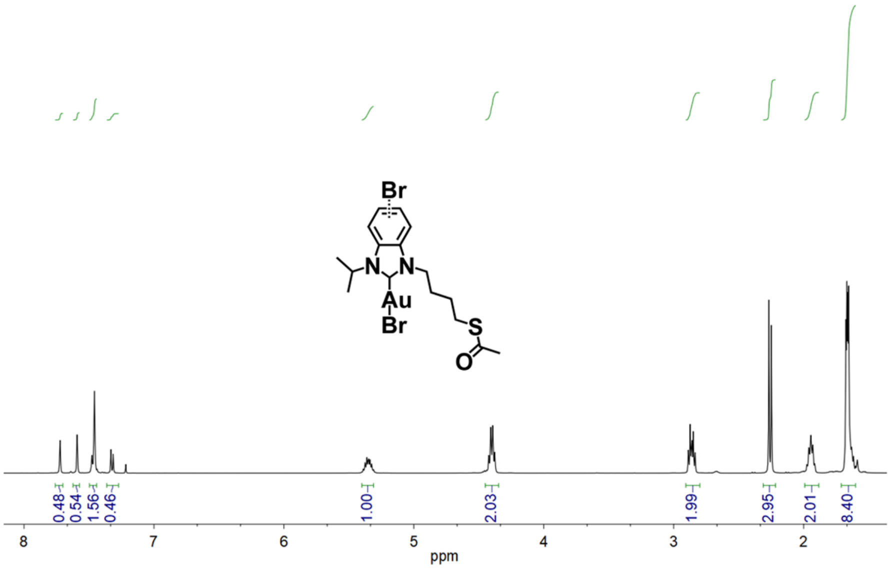

Figure S9. ^{1}H NMR spectrum of (**7**) in $CDCl_3$.

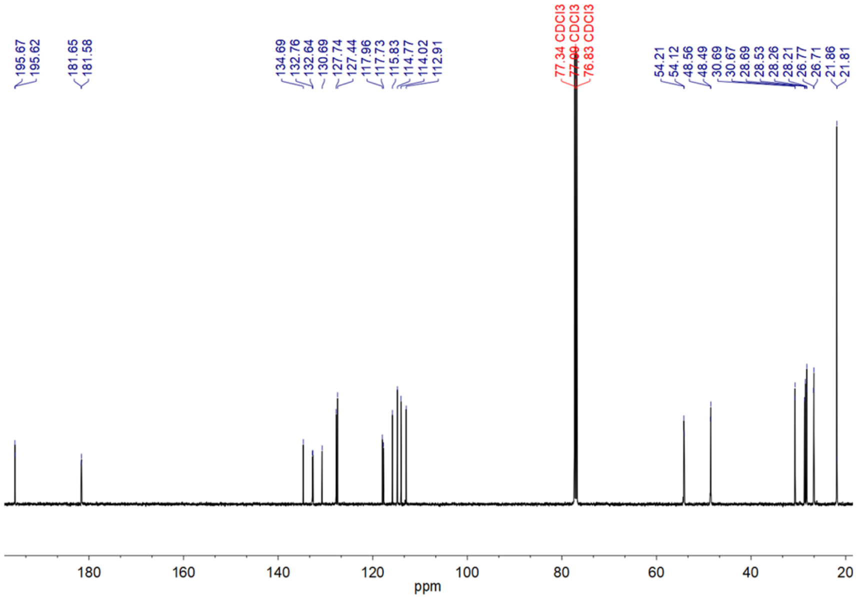

Figure S10. ^{13}C NMR spectrum of (**7**) in $CDCl_3$.

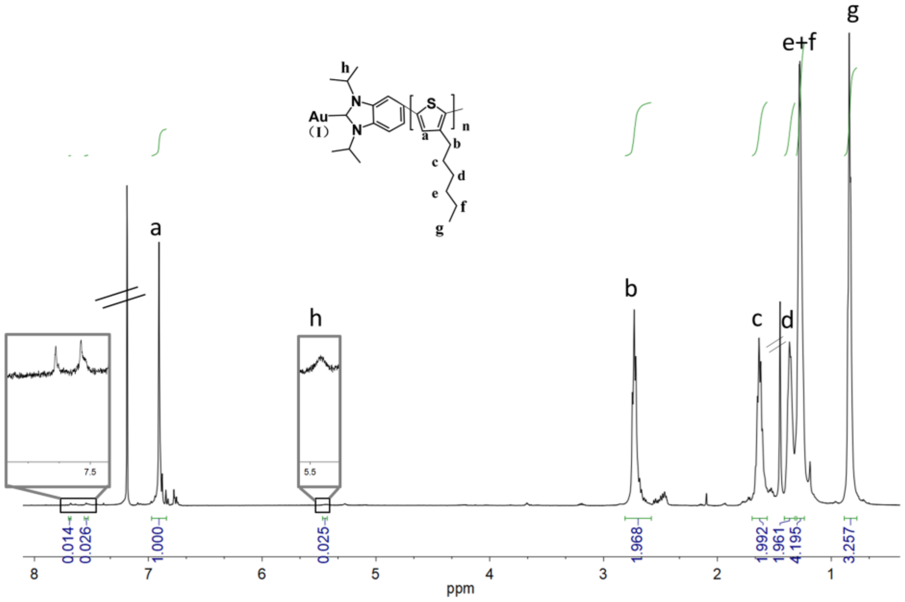

Figure S11. ^{1}H NMR spectrum of P3HT-NHC-Au in $CDCl_3$.

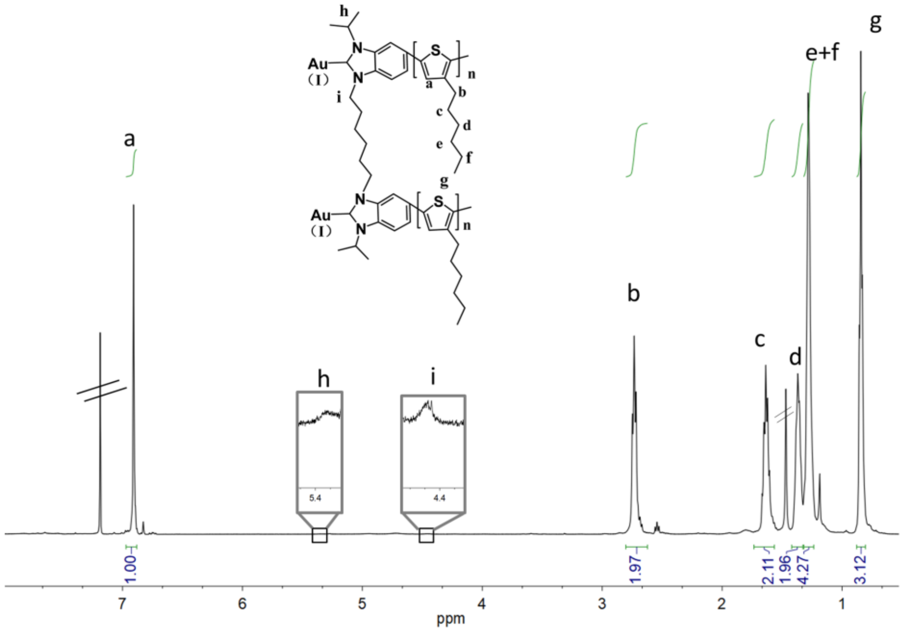

Figure S12. ^{1}H NMR spectrum of P3HT-DiNHC-Au in $CDCl_3$.

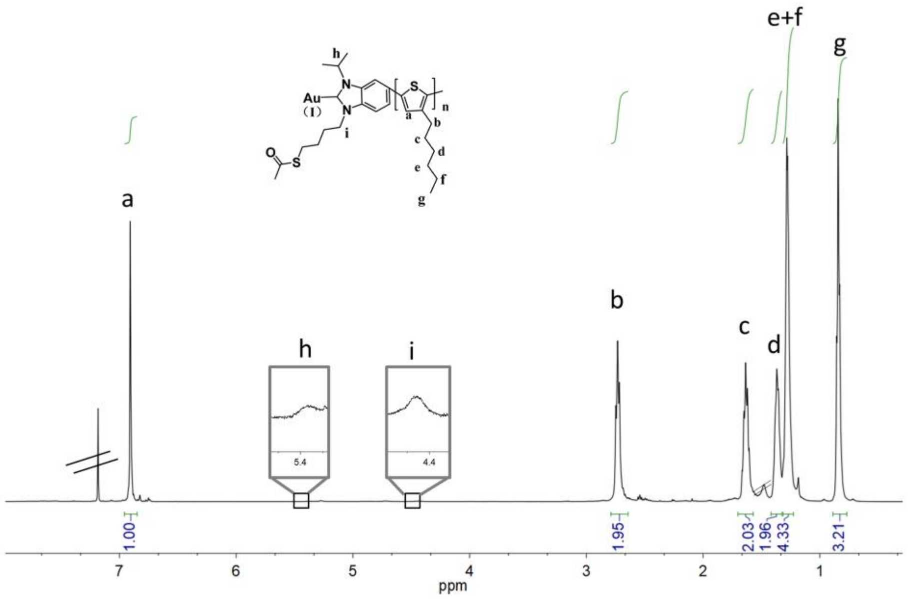

Figure S13. ^{1}H NMR spectrum of P3HT-SAcNHC-Au in $CDCl_3$.

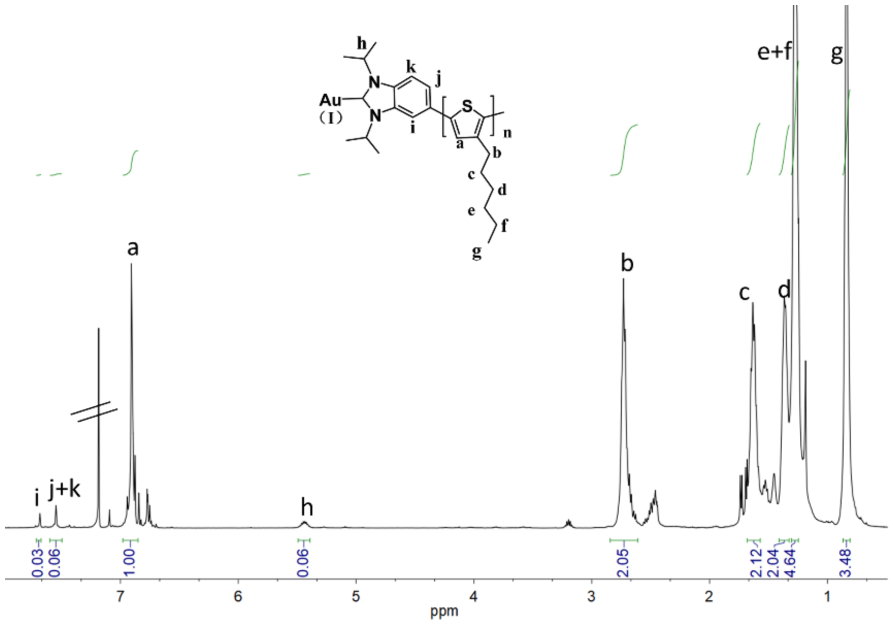

Figure S14. ^{1}H NMR spectrum of P3HT-NHC-Au-low in $CDCl_3$.

Gel Permeation Chromatography (GPC)

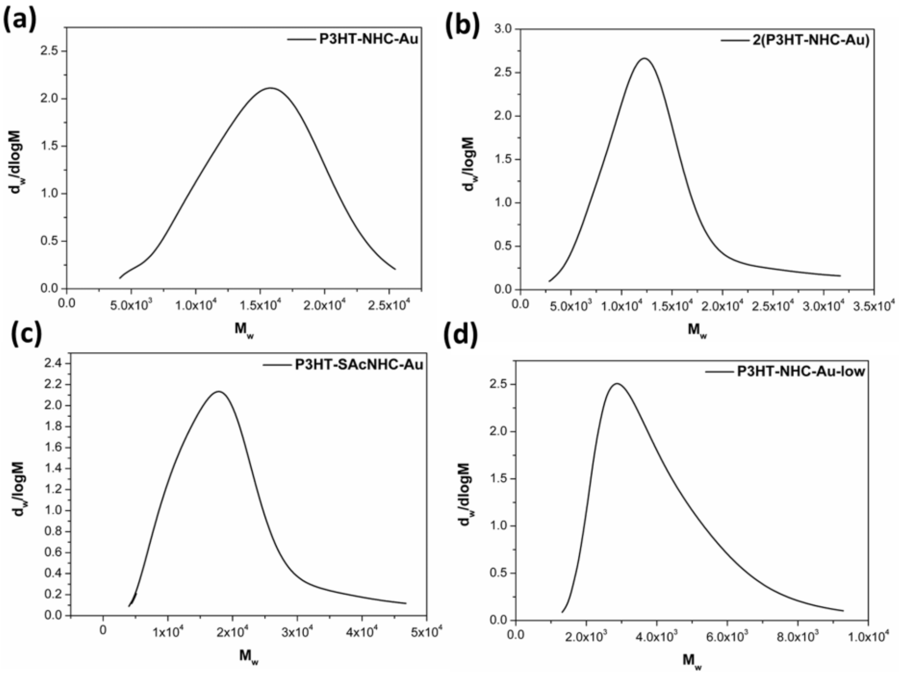

Figure S15. GPC curves of (a) P3HT-NHC-Au, (b) P3HT-DiNHC-Au, (c) P3HT-SAcNHC-Au and (d) P3HT-NHC-Au-low.

Table S1. Molecular weights of the polymers. M_w [g/mol], M_n [g/mol] and weight dispersity (PDI) as determined by GPC using a PS calibration standard.

	GPC[a]		
Samples	M_w	M_n	PDI
**P3HT-NHC-Au**	13200	11100	1.19
**P3HT-DiNHC-Au**	11500	9600	1.20
**P3HT-SAcNHC-Au**	15800	12800	1.23
**P3HT-NHC-Au-low**	2800	3200	1.14

[a] Relative to polystyrene standard, using chloroform as the eluent

MALDI-TOF

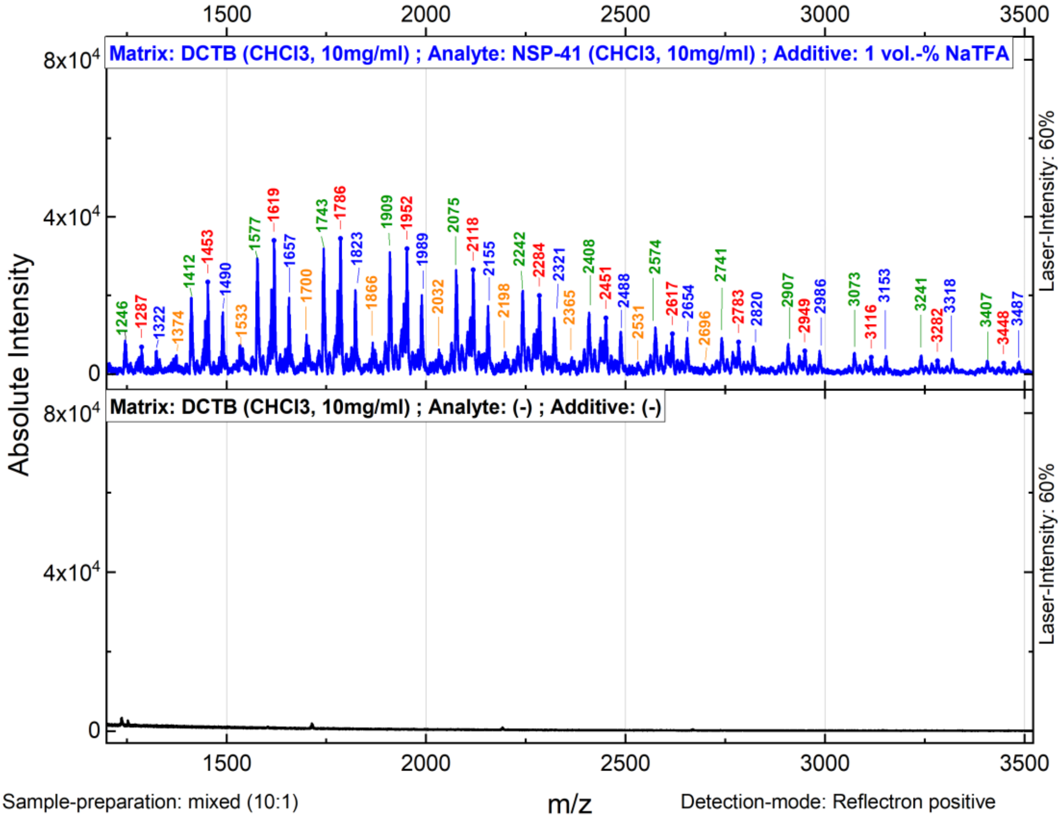

Figure S16 *top*: MALDI-TOF spectrum of P3HT-NHC-Au-low at 1/30 feed ratio. *bottom*: spectrum of matrix without polymer sample.

Table S2 Possible end-group combinations for the polymer NSP-41. [RU] is the repeating unit (166.1 g/mol).

Signal series	Possible combinations [Da]	1. measured signal [m/z]	Error [Da]
**1. (red)**	Na^+ + 9 x [RU] + H+ NHC-Au-Cl<br>=> 23.0 + 9 x 166.1 + 1.0 + 434.1= **1953**	1952	1
**2. (green)**	11 x [RU] + Br + H<br>=> 11 x 166.1 + 79.9 + 1.0 = **1908**	1909	1
**3. (blue)**	2 x Br + 11 x [RU]<br>=> 79.9 x 2 + 11 x 166.1 = **1986.9**	1989	2.1
**4. (orange)**	1 x Br + 9 x [RU] + NHC-Au-Cl + Na^+<br>=> 79.9 + 9 x 166.1 + 434.1 + 23 = **2031.9**	2032	0.1

X-ray Photoelectron Spectroscopy (XPS)

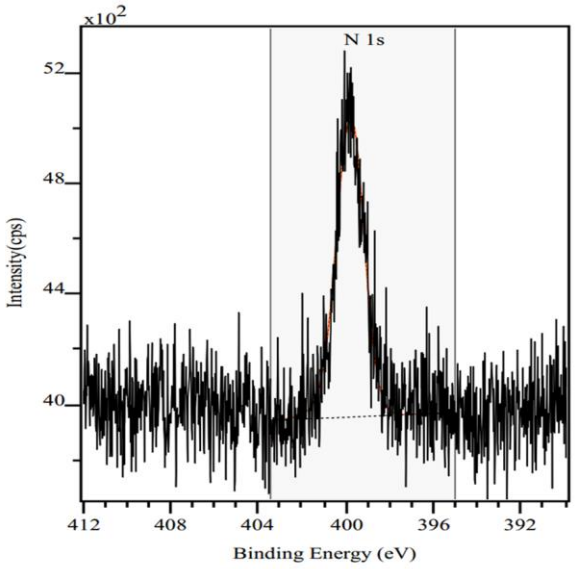

Figure S17. XPS N1s emission spectrum of P3HT-NHC@Au NP.

DFT

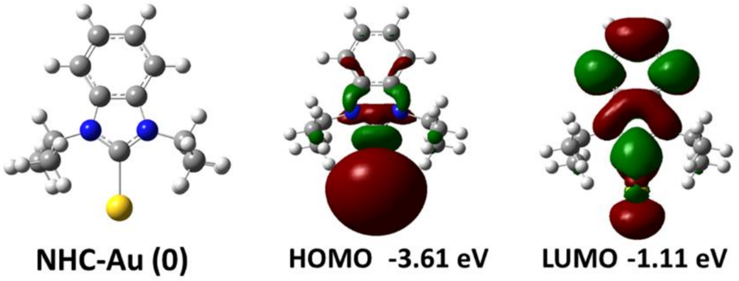

Figure S18. Molecular structure of NHC-Au (0), and HOMO and LUMO distributions and energy levels derived from DFT calculations (B3LYP/6-31G(d)).

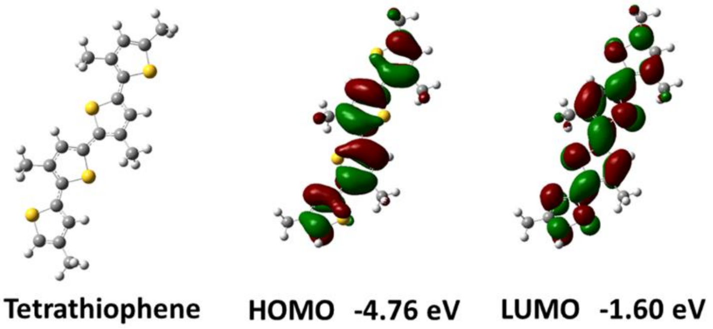

Figure S19. Molecular structure of tetrathiophene, and HOMO and LUMO distributions and energy levels derived from DFT calculations (B3LYP/6-31G(d)).

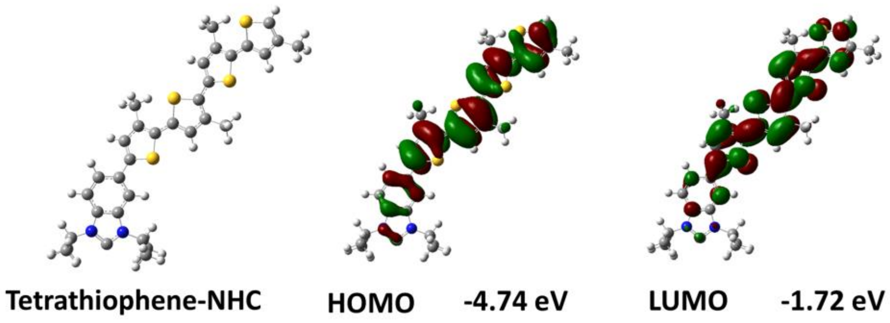

Figure S20. Molecular structure of tetrathiophene-NHC, and HOMO and LUMO distributions and energy levels derived from DFT calculations (B3LYP/6-31G(d)).

References

[S1] G. Wilke, G. Herrmann *Angew. Chem., Int. Ed.* **1966**, *5*, 581.

[S2] C. M. Crudden, J. H. Horton, I. I. Ebralidze, O. V Zenkina, A. B. McLean, B. Drevniok, Z. She, H.-B. Kraatz, N. J. Mosey, T. Seki, Seki, E. C. Keske, J. D. Leake, A. Rousina-Webb and G. Wu, *Nat. Chem.* **2014**, *6*, 409

[S3] J. F. DeJesus, M. J. Trujjllo, J. P. Camden, D. M. Jenkins, *J. Am. Chem. Soc*. **2018**, *140*, 1247.

[S4] V. Senkovskyy, R. Tkachov, T. Beryozkina, H. Komber, U. Oerte, M. Horecha, V. Bocharova, M. Stamm, S. A. Gevorgyan, F. C. Krebs, A. Kiriy, *J. Am. Chem. Soc*. **2009**, *131*, 3505.

[S5] A. M. Steiner, M. Mayer, M. Seuss, S. Nikolov, K. D. Harris, A. Alexeev, C. Kuttner, T. A.F. König, A. Fery, *ACS Nano* **2017**, *11*, 8871.
